# Shape Memory Properties of PBS-Silica Hybrids

**DOI:** 10.3390/ma7020751

**Published:** 2014-01-27

**Authors:** Katia Paderni, Paola Fabbri, Maurizio Toselli, Massimo Messori

**Affiliations:** 1 Dipartimento di Ingegneria “Enzo Ferrari”, Università di Modena e Reggio Emilia, via Vignolese 905/A, Modena 41125, Italy; E-Mails: katia.paderni@unimore.it (K.P.); paola.fabbri@unimore.it (P.F.); 2 Dipartimento di Chimica Industriale “Toso Montanari”, Università di Bologna, Viale Risorgimento 2, Bologna 40136, Italy; E-Mail: maurizio.toselli@unibo.it; 3 Consorzio Interuniversitario per la Scienza e Tecnologia dei Materiali (INSTM), Via G. Giusti 9, Firenze 50121, Italy

**Keywords:** poly(butylene succinate) (PBS), organic/inorganic, shape memory polymers, crosslinking

## Abstract

A series of novel Si–O–Si crosslinked organic/inorganic hybrid semi-crystalline polymers with shape memory properties was prepared from alkoxysilane-terminated poly(butylene succinate) (PBS) by water-induced silane crosslinking under organic solvent-free and catalyst-free conditions. The hydrolyzation and condensation of alkoxysilane end groups allowed for the generation of silica-like crosslinking points between the polymeric chains, acting not only as chemical net-points, but also as inorganic filler for a reinforcement effect. The resulting networks were characterized using differential scanning calorimetry (DSC), thermogravimetric analysis (TGA), dynamic-mechanical analysis (DMA) and tensile and shape memory tests to gain insight into the relationship between the polymeric structure, the morphology and the properties. By controlling the molecular weight of the PBS precursor, a fine tuning of the crosslinking density and the inorganic content of the resulting network was possible, leading to different thermal, mechanical and shape memory properties. Thanks to their suitable morphology consisting of crystalline domains, which represent the molecular switches between the temporary and permanent shapes, and chemical net-points, which permit the shape recovery, the synthesized materials showed good shape memory characteristics, being able to fix a significant portion of the applied strain in a temporary shape and to restore their original shape above their melting temperature.

## 1. Introduction

Due to increasing concerns for sustainable development and the impact of materials on the environment, bio-degradable and bio-based polymers have attracted intensive interest in the past few decades [1]. Poly(butylene succinate) (PBS) is one of the few commercialized bio-compostable polymers with balanced performance in thermal and mechanical properties, as well as thermoplastic processability compared to other common plastics [2–4]. PBS is a highly crystalline polyester, with its melting point at around 115 °C, and currently, commercial PBS is mainly obtained from the polymerization of petroleum-based succinic acid and 1,4-butanediol; however, the two monomers can be also derived from renewable resources via fermentation [5,6]. As a result, PBS becomes a very promising material as a substitute for conventional plastics [7,8].

**Scheme 1.**
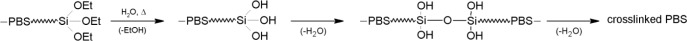
Crosslinking reactions of alkoxysilane-terminated poly(butylene succinate) (PBS).

Shape memory polymers (SMPs) are a class of smart materials that have the ability to retain a temporary shape and to return to their original shape with the use of a suitable trigger, typically an increase in temperature [9]. This unique capability makes SMP very attractive for different fields of applications, such as biomedical devices, aerospace, textiles, energy, bionics engineering, electronic engineering, civil engineering and household products [10]. The key structural elements of thermally activated SMPs are switching domains related to a thermal transition (glass transition or melting temperature), which are responsible for the fixation of a temporary shape, and net-points, which are responsible for effectively restoring the permanent shape and are typically represented by physical or chemical crosslinking [11–14]. Concerning a possible way of chemical crosslinking, silane grafting or copolymerization or endcapping and subsequent water-crosslinking of polymers have received much attention in recent years, not only for industrial applications, but also in fundamental research, because of various advantages, such as easy processing, low capital investment and the favorable properties of the processed materials [15,16]. In these processes, silanes are first incorporated into a polymer backbone or bonded as terminal groups of the polymer, followed by hydrolysis (the formation of –Si–OH groups) and condensation, leading to the formation of siloxane (Si–O–Si) linkages between polymer chains [17]. A proposed mechanism of a water-induced crosslinking process for alkoxysilane-terminated PBS is shown in Scheme 1.

In this scenario, the organic-inorganic hybrid materials have recently received increasing attention in the field of SMPs, because of their improved mechanical properties together with the possibility of easy and fine tuning of the shape memory performance through the modification of suitable structural factors of the networks [18–21].

Considering the scientific interest towards environmentally friendly polymers, shape-memory properties and the water-induced crosslinking technique, in this paper, a series of crosslinked PBS-silica networks was prepared using water-induced silane crosslinking, starting from alkoxysilane-terminated PBS. The crosslinked materials, having Si–O–Si domains acting not only as net-points, but also as inorganic filler for reinforcement, were investigated for their molecular architecture and characterized in terms of thermal properties, dynamic-mechanical properties and shape-memory behavior, exploiting their melting temperature for the activation of the recovery process.

## 2. Experimental

### 2.1. Materials

Succinic acid (SA), 1,4-butanediol (BDO), titanium (IV) butoxide [Ti(OBu)_4_, 3-(triethoxysilyl)propyl isocyanate] (ICPTS), water (H_2_O) and chloroform (CHCl_3_) were the high purity reagents purchased from Sigma-Aldrich (Milan, Italy) and used as received without any further purification.

### 2.2. Synthesis and Molecular Characterization of α,ω-Hydroxyl-Terminated Poly(butylene succinate)

**Scheme 2.**
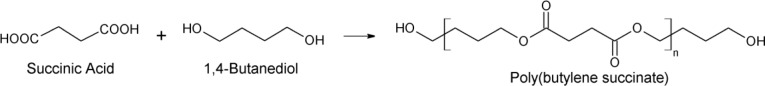
Synthesis of α,ω-hydroxyl-terminated poly(butylene succinate).

α,ω-Hydroxyl-terminated PBSs were synthesized by melt polycondensation from 1 mol SA and 1.2 mol BDO (Scheme 2) in the presence of Ti(OBu)_4_ catalyst at 190 °C for 3 h under nitrogen flow and followed by 230 °C under reduced pressure for predetermined times in order to obtain PBS diol with different average-number molecular weights (*M_n_*). The obtained materials were coded as PBS_x, as reported in Table 1.

**Figure 1.**
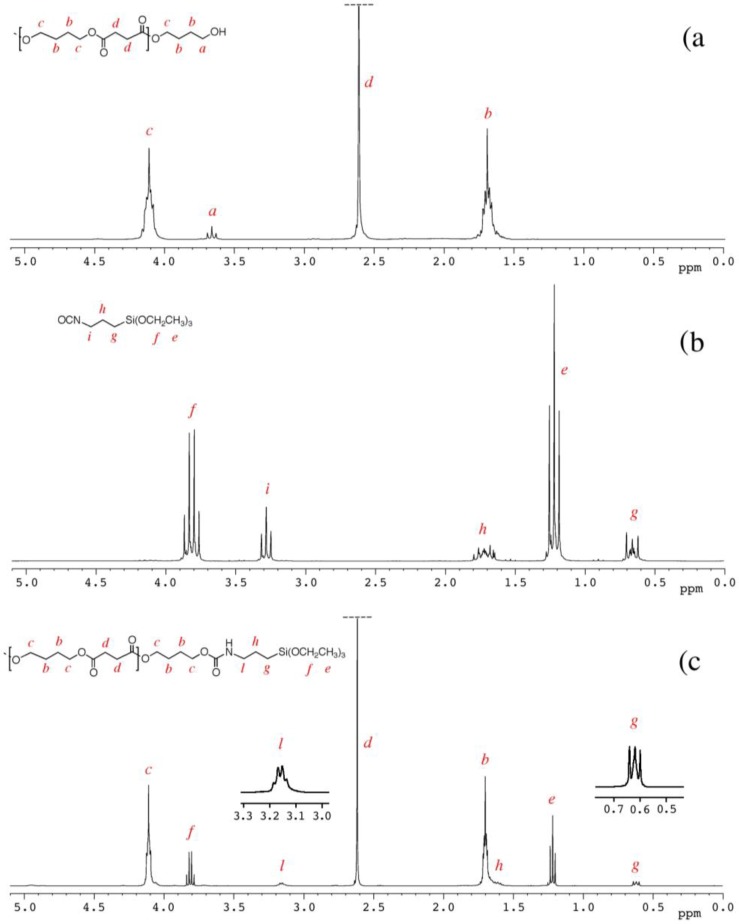
^1^H NMR spectra of (**a**) α,ω-hydroxyl-terminated PBS_2; (**b**) 3-(triethoxysilyl)propyl isocyanate (ICPTS); and (**c**) α,ω-triethoxysilane-terminated PBS_2_Si.

The degree of polymerization (*D_P_*) and the derived molecular weight were determined by ^1^H NMR spectra, considering the intensities at 3.65 ppm (*I*_3.65_), attributable to the methylene group linked with the terminal hydroxyl group of the PBS molecular chain (signal *a* in Figure 1) and 4.10 ppm (*I*_4.10_), attributable to the methylene protons in the repeating units of PBS (signal *c* in Figure 1), by the following equations:

$$D_{p}=\frac{I_{4.10}}{I_{3.65}}$$ (1)

$$M_{n}=172\times D_{P}+90$$ (2)

where 172 and 90 are the molecular weights of the repeating unit of PBS and of the terminal unit of BDO, respectively.

### 2.3. Synthesis and Molecular Characterization of α,ω-Triethoxysilane-Terminated Poly(butylene succinate)

**Scheme 3.**
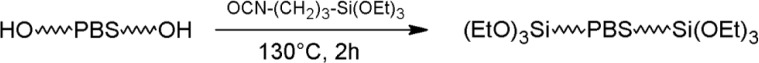
Preparation of α,ω-triethoxysilane-terminated poly(butylene succinate).

PBS_x samples were dried overnight at 90 °C under dynamic vacuum in the presence of molecular sieves just before reaction with ICPTS. Dried PBS_x and ICPTS were introduced into a glass flask, previously flushed three times with cycles of vacuum-nitrogen. The reactions were carried out in bulk at 130 °C for 2 h under a nitrogen atmosphere and magnetic stirring. ICPTS was added with a 20% stoichiometric excess with respect to the hydroxyl groups of PBS (Scheme 3).

Unreacted ICPTS was removed by dynamic vacuum at the end of the reaction. The obtained materials were coded as PBS_x_Si.

The ^1^H NMR spectrum of PBS_2_Si with the corresponding signals assignment, reported in Figure 1c, shows that the signal at 3.65 ppm (related to the methylene groups adjacent to the hydroxyl end groups of PBS, signal *a*) has a very low intensity, and the signal at 3.15 ppm (related to the methylene groups adjacent to the nitrogen atom of the urethane group, signal *l*) is shifted and modified in its shape with respect to the parental signal, *i*, of ICPTS in Figure 1b (at 3.3 ppm and related to the methylene groups adjacent to the nitrogen atom of the isocyanate group).

Finally, the degree of silanization (*D_SI_*) of the triethoxysilane-terminated PBS was calculated considering the signal at 3.15 ppm (related to the properly end-capped groups of PBS) and the one at 3.65 ppm (related to the groups of hydroxyl-terminated PBS), according to the following equation:

$$D_{SI}=\frac{I_{3.15}}{I_{3.15}+I_{3.65}}$$ (3)

### 2.4. Crosslinking of α,ω-Triethoxysilane-Terminated Poly(butylene succinate)

The subsequent crosslinking of triethoxysilane-terminated PBS was carried adding 15 wt% of H_2_O to powdered PBS_x_Si. Silicone molds having dimensions of 80 × 5 × 3 mm^3^ were filled with the aforementioned wet PBS and heated at 140 °C. After complete melting of the PBS, dynamic vacuum was applied for 10 min to eliminate air bubbles eventually present in the sample. Then, the samples were cured at this temperature for 6 h to promote the crosslinking of PBS, induced by the formation of silica domains after hydrolysis and the condensation reactions of alkoxysilane end groups (Scheme 1). In order to increase the extent of the condensation reactions and the gel content, a post-curing was carried out for 8 h at 160 °C. The obtained materials were coded as X-PBS_x.

### 2.5. Degree of Swelling and Gel Content

After curing, specimens having a rectangular shape (a length of 20 mm and a cross-section of 5 × 1 mm^2^) and initial weight *m*_0_ were placed in 20 mL of CHCl_3_ at room temperature. The swollen specimens at equilibrium swelling were weighed to determine *m_s_* (approximately after 24 h), and they were subsequently dried at room temperature until a constant weight in order to determine the residual weight after extraction (*m_d_*).

The degree of swelling (*Q*) and the gel content (*G*) were calculated according to the following equations [22]:

$$Q=1+\frac{\rho_{1}}{\rho_{2}}\left(\frac{m_{s}}{m_{d}}-1\right)$$ (4)

$$G=\frac{m_{d}}{m_{0}}$$ (5)

where ρ_1_ and ρ_2_ are the densities of the swelling solvent and PBS, respectively. A density of 1.26 g·cm^−3^ was used for ρ_2_ [5], whereas the density of CHCl_3_ was taken as 1.48 g·cm^−3^. The degree of swelling of PBS was calculated, normalizing the calculated *Q* to the actual PBS weight fraction (taking into account the presence of silica).

### 2.6. Determination of the Crosslinking Density

Crosslinking density, defined as moles of effective network chains per unit volume, was computed according to the Flory–Rehner equation [23] from equilibrium swelling experiments after estimating the volume fraction, *υ*_2_, of the organic phase in the swollen specimens:

$$\nu =\frac{-[\ln(1-\upsilon_{2})+\upsilon_{2}+\chi_{1-2}\cdot \upsilon_{2}^{2}]}{V_{s}\cdot \left(\upsilon_{2}^{1/3}-\frac{2\upsilon_{2}}{f}\right)}=\frac{\rho_{2}}{M_{c}}$$ (6)

where *V_s_* is the molar volume of the solvent, *f* is the functionality of the networks junctions, χ_1−2_ is the Flory–Huggins interaction parameter between the solvent and polymer and *M_c_* is the average molecular weight between crosslinks [7]. The calculation of crosslinking density is detailed in the Supplementary Information.

### 2.7. Instrumental Analysis

^1^H NMR spectra were recorded in CDCl_3_ at 400 MHz with a Bruker spectrometer AVANCE-400 (Bruker Corp., Billerica, MA, USA). Chemical shifts were referred to tetramethylsilane at 0 ppm.

FT-IR spectroscopy was performed by using an Avatar 330 FT-IR Thermo Nicolet spectrometer (Thermo Fisher Scientific, Inc., Madison, WI, USA) operating in the Attenuated Total Reflection (ATR) mode. Thirty-two scans with a resolution of 4 cm^−1^ were used for each recorded spectrum.

Thermogravimetric analysis (TGA) was performed by a Perkin-Elmer TGA7 thermogravimetric analyzer (PerkinElmer, Inc., Waltham, MA, USA) under nitrogen flow, from 30 up to 750 °C at a heating rate of 10 °C min^−1^.

Differential scanning calorimetry (DSC) was performed on a TA DSC 2010 (TA Instruments, New Castle, DE, USA) purged with nitrogen. Generally, if not otherwise specified, the samples were firstly heated at 150 °C at 5 °C·min^−1^ and kept at this temperature for 2 min to erase the thermal history. Thermograms were recorded at a heating/cooling rate of 5 °C·min^−1^ over the range −30/150 °C. The degree of the crystallinity of PBS was calculated by considering a melting enthalpy of 210 J·g^−1^ for the 100% crystalline PBS [24], referring to the measured melting enthalpy for the crosslinked samples to the actual PBS weight fraction (taking into account the presence of silica).

Dynamic-mechanical thermal analysis (DMTA) was carried out on rectangular strips (average length: 16 mm; average cross-section: 5 mm^2^) by means of a TA DMA Q800 (TA Instruments, New Castle, DE, USA), by employing a tensile configuration. The specimens were tested at a frequency of 1 Hz, under a displacement amplitude of 3 μm and a tensile preload of 0.01 N. The tests were carried out by heating the specimens at 3 °C·min^−1^ from −50 to 150 °C. The mechanical response of the materials above the melting temperature in the rubbery region at *T*_high_ (with *T*_high_ = *T*_efg_ + 8 °C, *T*_efg_ is the extrapolated end temperature in the storage modulus drop in correspondence with the melting transition) was investigated by subjecting rectangular strips, such as those described above, to tensile tests, carried out in the DMA Q800 under the stress control module at a testing rate of 10^−2^ MPa·min^−1^. For the determination of mechanical properties, at least three samples were tested, and values are given as the average ± standard deviation.

### 2.8. Shape Memory Behavior

The shape memory behavior of the samples was investigated by the application of properly designed stress-controlled thermo-mechanical histories, carried out using the aforementioned DMA machine, employed under tensile configuration on rectangular strip specimens, such as those described above.

The shape memory cycles were performed by applying an early “programming” step and a subsequent recovery step and repeating them three times. The “programming” step was carried out by deforming the specimens above their melting temperature and cooling them below their crystallization temperature under fixed stress conditions. The specimens were heated at 5 °C·min^−1^ to *T*_high_ (defined above in the Instrumental Analysis subsection), deformed under a ramp of 10^−2^ MPa·min^−1^ up to a stress of 0.15 or 0.25 Mpa and cooled at 5 °C·min^−1^ to *T*_low_ = 27 °C, while maintaining constant the stress level attained during the loading step. The recovery behavior was investigated by heating the deformed specimens above the melting temperature under quasi stress-free conditions, while monitoring the strain evolution as a function of temperature (the specimens were heated at 5 °C·min^−1^ up to *T*_high_, under the application of a small stress of 10^−4^ Mpa, which allowed for continuous tracking of the specimen length). At the end of each heating/cooling segment, an isothermal step of 20 min was applied to equilibrate the sample temperature. The shape fixity ratio (*R_f_*) and the shape recovery ratio (*R_r_*) were determined from the following equations:

$$R_{f}(N)=\frac{\varepsilon_{u}(N)}{\varepsilon_{l}(N)}$$ (7)

$$R_{r}(N)=\frac{\varepsilon_{l}(N)-\varepsilon_{p}(N)}{\varepsilon_{l}(N)-\varepsilon_{p}(N-1)}$$ (8)

where ε*_u_* (*N*) is the tensile strain after unloading and ε_l_ (*N*) is the maximum strain achieved under fixed stress after cooling to *T*_low_ of the *N-*th cycle and ε*_p_* (*N*) and ε*_p_* (*N* – 1) are the residual strains after recovery in the *N-*th and in the previous cycle, respectively [25].

## 3. Results and Discussion

### 3.1. Synthesis of α,ω-Hydroxyl-Terminated and α,ω-Triethoxysilane-Terminated Poly(butylene succinate)

PBS_x were prepared by the polycondensation of BDO with SA using Ti(Obu)_4_ as a catalyst (Scheme 2). In order to obtain α,ω-hydroxyl-terminated PBS, the feed molar ratio of BDO to SA was fixed at 1.2:1. The molecular weights of PBS diols (shown in Table 1) were varied by varying the polycondensation time [22], and they were determined by ^1^H NMR spectroscopy (Figure 1a) calculating the degree of polymerization (*D_p_*) as described in the Experimental Section.

Indeed, triethoxysilane end-capped PBSs can be easily prepared by bulk reaction of α,ω-hydroxyl-terminated PBS with ICPTS. ^1^H NMR spectra of PBS_2_Si and of the starting materials (PBS_2 and ICPTS) are reported in Figure 1 and discussed in the Experimental Section.

The results (Table 1) show an almost complete reaction of the hydroxyl end groups of PBS, with the formation of urethane groups attributable to triethoxysilane terminal groups, with *D_SI_* in the range 91%–98%.

**Figure 2.**
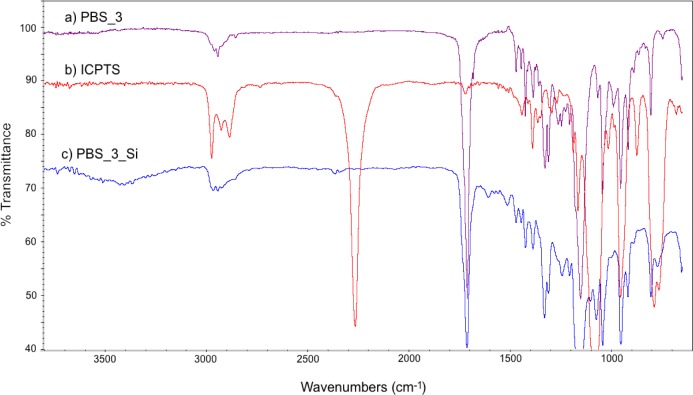
FT-IR spectra of (**a**) α,ω-hydroxyl-terminated PBS_3; (**b**) ICPTS; and (**c**) α,ω-triethoxysilane-terminated PBS_3_Si.

FT-IR spectra of PBS_3, ICPTS and PBS_3_Si are reported in Figure 2. In Figure 2c, the absence of the sharp peak corresponding to isocyanate groups (at 2275 cm^−1^) and the presence of the absorption bands of the urethane group (in the range 3200–3500 cm^−1^ for NH stretching and 1650–1515 cm^−1^ for NH bending) [26] with respect to the spectra of the reactants (Figures 2a,b) are in agreement with the expected molecular structure. Similar spectra were obtained also for the other PBSs, both for ^1^H NMR and for FT-IR.

### 3.2. Degree of Swelling, Gel Content and Crosslinking Density of Crosslinked PBSs

α,ω-Triethoxysilane-terminated PBSs were allowed to undergo the silane-crosslinking process as described in the Experimental Section. The silane-end capped polymers were crosslinked in the melt state in presence of water. The crosslinking reaction involves the hydrolysis of the alkoxy groups with H_2_O to give silanols (–Si–OH), followed by the condensation of the formed hydroxyl groups to generate stable siloxane linkages (Si–O–Si) between polymer chains, leading to the formation of three-dimensional networks [27] (Scheme 1).

Crosslinking densities were determined by the Flory–Rehner equation, and the gel content and degree of swelling were determined according to the procedure described in the Experimental Section and are reported in Table 2. Extended crosslinking occurred in all the samples, as attested by the high gel content (*G* = 91.5%–99.2%), indicating a high curing efficiency during the crosslinking step. As expected, the degree of swelling increases as the crosslinking density decreases and, therefore, by increasing the molecular weight of the PBS precursor (*Q* = 4.9–9.8). Indeed, it is possible to obtain an easy control of the crosslinking density, in the range between 2.52 × 10^−4^ and 9.44 × 10^−4^ mol·cm^−3^, by changing the molecular weight of the PBS precursor, which represents a key parameter for controlling not only the crosslinking density of polymer network, but also the other properties, such as the thermal-mechanical and shape memory ones, as discussed in next sections. The average molecular weight between crosslinking points (*M_c_*) is very close to the molecular weight of the PBS precursor, confirming that hydrolysis and condensation successfully occurred among the different triethoxysilane end-groups, giving rise to condensed silica domains, which act as crosslinks among different PBS chains.

It has to be emphasized indeed that silane crosslinking has been achieved, avoiding some disadvantages, such as thickness limitation and residual monomers typically used in radiation crosslinking, the risk of pre-curing and the use of potentially toxic components, such as organic peroxides in radical crosslinking [28,29].

### 3.3. Thermogravimetric Analysis

**Figure 3.**
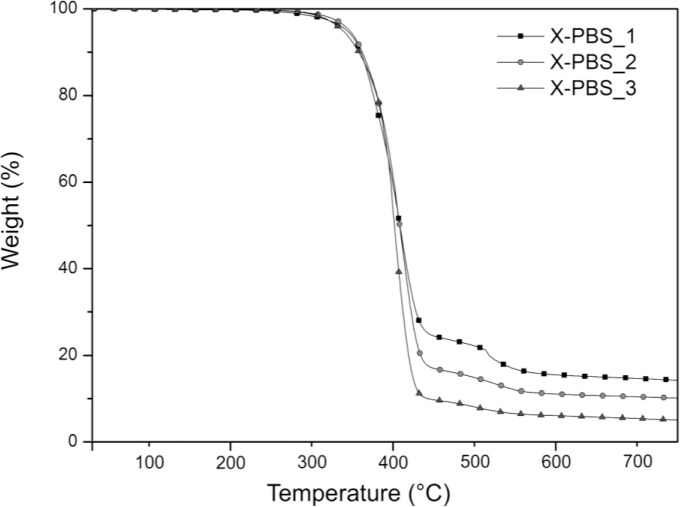
Thermogravimetric analysis (TGA) curves of alkoxysilane crosslinked PBSs.

The thermogravimetric analysis curves of crosslinked PBSs are summarized in Figure 3 and Table 3. Increasing the molecular weight of the PBS precursor, the residual mass decreases from 14.1% to 4.9%, indicating a lower silica content generated, starting from the hydrolysis and condensation reactions of the triethoxysilane terminal groups, whose concentration is lower for the highest molecular weight.

The main degradation step starts around 300 °C and ends around 460 °C for all the materials, and it could be attributable to the decomposition of the molecule chains far away from the crosslinking points, while the drop occurring at higher temperatures (in the range 460–580 °C) may be related to the decomposition of the molecular segments next to the crosslinking points [2], which face a stronger restriction. For X-PBS_1, the concentration of these molecular segments around the crosslinking points is higher in comparison with the other crosslinked PBS, and this justifies a more pronounced weight drop. Evaluating the temperature at which the decomposition rate is maximum (*T*_max_) from the DTGA curves, a slight decrease of 11 °C is observed, increasing the molecular weight: this could be attributable to a slightly higher thermal stability for X-PBS_1, due to a higher crosslinking density and a higher silica content.

### 3.4. Thermal Properties Evaluated by DSC

**Figure 4.**
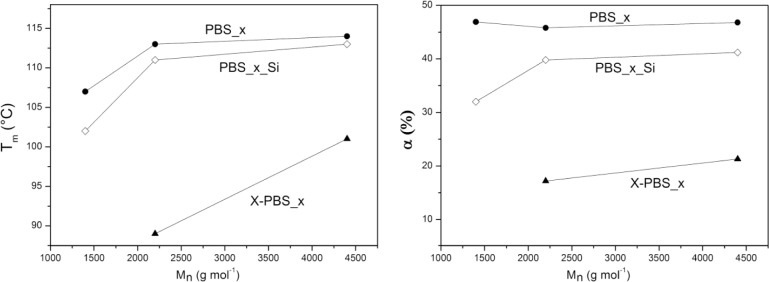
Melting temperature (*T_m_*) and degree of crystallinity (α) of PBS_x, PBS_x_Si and X-PBS_x *versus* the molecular weight of the PBS diol.

Thermal properties (crystallization temperature, *T*_c_; melting temperature, *T_m_*; degree of crystallinity, α) of α,ω-hydroxyl-terminated PBSs, α,ω-triethoxysilane-terminated PBSs and crosslinked PBSs determined by DSC are reported in Table 4 and Figure 4. The thermal properties are strongly dependent on the molecular weight of the PBS precursors: the higher the *M_n_*, the higher the thermal transitions and the degree of crystallinity, except for the crystallinity content in the hydroxyl-terminated PBSs, which is more or less the same for all three samples.

In particular, α,ω-hydroxyl-terminated PBS (PBS_x) showed increasing *T_m_* values by increasing the molecular weight from a minimum of 107 °C for PBS with molecular weight equal to 1400 g·mol^−1^ to a maximum of 114 °C for PBS with a higher molecular weight, representing also the value of the high-molecular weight PBS [1,5], while *T_c_* varies from a minimum of 69 °C to a maximum of 77 °C. Both *T_m_* and *T_c_* values show little change for macromolecules with a higher molar mass, whereas lower mass ones change faster [30].

The introduction of triethoxysilane terminal groups depressed the crystallization and melting temperatures and the degree of crystallinity compared with the corresponding α,ω-hydroxyl-terminated PBS and with a stronger effect for the lowest molecular weight (PBS_1_Si), indicating that the presence of bulky terminal groups in the PBS chain negatively affects the crystallization process.

After crosslinking, *T_c_* and *T_m_* values were even lower than the corresponding PBS_x_Si. The network with the lowest PBS segment length (X-PBS_1) showed a very broad melting transition (*T_m_* = 60 °C and α = 15%) only in the very first heating scan (that is, without erasing the thermal history). No melting transition has been observed in the subsequent heating scan after a cooling rate of 5 °C·min^−1^ from 150 °C, indicating an inhibition of the crystallization process, due to the restrictions imposed by the highly crosslinked structure. Considering the other two crosslinked materials, the transition temperatures decreased much more for X-PBS_2 passing from alkoxysilane-terminated to crosslinked PBSs: a *T_c_* decrease of 19 °C and a *T_m_* decrease of 22 °C; and the degree of crystallinity became less than half (from 39.8% to 17.2%). This phenomenon could be ascribed to a more hindered crystallization, due to the higher restrictions imposed by a more tightly crosslinked network and to a higher content of inorganic Si–O–Si domains. This restriction effect is less significant in X-PBS_3, having a longer molecular segment length between crosslinking points. The present data reflect the same behavior already observed for crosslinked polycaprolactone (PCL) [22,28] in terms of the dependence of thermal properties on the molecular weight of PCL precursors, on the end-group modification and crosslinking, and the disappearing of crystallization for tightly crosslinked networks (PCL molecular weights lower than 2000 g·mol^−1^).

It is thus clearly evidenced that it is possible to vary the characteristic temperatures of the shape memory process and the material crystallinity content by changing the molecular weight of the PBS precursor.

### 3.5. Dynamic-Mechanical Thermal Analysis

**Figure 5.**
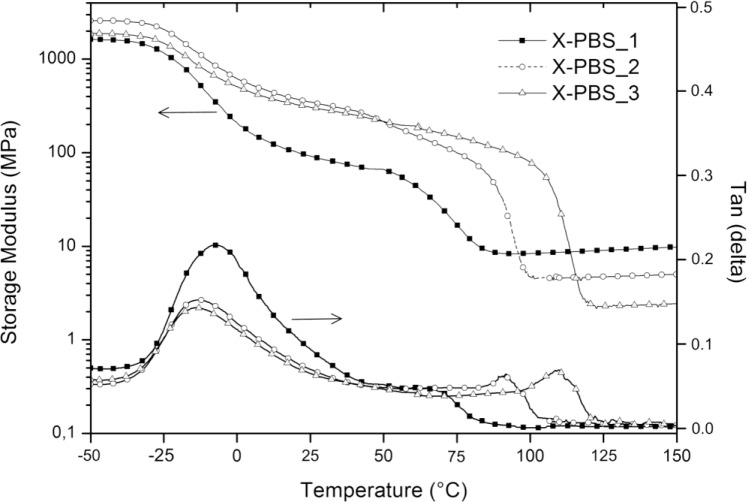
Dynamic-mechanical thermal properties of crosslinked PBS.

Figure 5 shows the storage modulus (E’) and loss factor (tanδ) of crosslinked PBSs, and the obtained results are summarized in Table 5. It can be clearly seen that the *T*_g_, evaluated from the peaks of the tanδ curves, increases with an increase in the crosslinking density from −14 °C for the lowest crosslinked material to −8 °C for the highest crosslinked one. The increasing of crosslinking junctions between polymer chains restricts segmental mobility, and the restriction on motion of PBS macromolecules would increase the energy requirements for the transition and, thereby, raise *T_g_* [16]. Evaluating tanδ, which represents a measure of the energy dissipation, it could be observed that the intensity in the correspondence of the peak (tanδ_max_) decreases, increasing the molecular weight of the PBS precursor, and this could be mainly ascribed to a higher crystallinity content of X-PBS_2 and X-PBS_3 and a consequently lower damping behavior.

All materials exhibit the behavior typical of crosslinked semi-crystalline polymeric networks with respect to storage modulus curves. There is a glass state at low temperatures, with E’ staying at a high modulus plateau, the first drop related to the glass transition between −25 and 0 °C, the second sigmoidal drop due to the melting of crystalline domains and a rubbery state with a low E’ at high temperatures. The storage modulus was evaluated at three different temperatures: below the glass transition at −50 °C (E’_−50°C_), at room temperature (E’_27°C_) and in the rubbery region (E’_rubber_) at a temperature exceeding 8 °C, the extrapolated end temperature, which is the intersection of the inflectional tangent with the tangent extrapolated from temperatures above the melting transition. The storage modulus values at −50 and at 27 °C show a maximum corresponding to X-PBS_2, and this behavior could be explained taking into account that E’ below *T_m_* is mainly dependent on silica content (which increases by decreasing the molecular weight of PBS precursor) and on the degree of crystallinity of the polymeric phase (which increases by increasing the molecular weight of the PBS precursor), thus resulting as highest for the material with intermediate values for these two properties. Concerning the melting transition, the beginning of the modulus drop occurs at higher temperatures for less crosslinked materials, reflecting the same trend already observed with DSC, and the drop is proportional to the crystallinity content. In the rubbery region, the storage modulus is related to the crosslinking density and the inorganic content, varying from 8 MPa for the highest crosslinked material also with the highest silica content to 2 MPa for the lowest crosslinked one with the lowest silica content. Considering the theory of rubber elasticity, the crosslinking density (*νe*) was determined by the following equation:

$$E^{\prime }=3\nu_{e}RT$$ (9)

Where E’ is the storage modulus of the crosslinked polymer in the rubbery plateau region above *T_m_* (E’_rubber_), *R* is the gas constant (8.314 J·K^−1^·mol^−1^) and T is the absolute temperature (K) [17]. The obtained results, reported in Table 5, confirm the expected trend, with a higher crosslinking density for X-PBS_1 having the lowest PBS precursor molecular weight with a crosslinking density of 8.76 × 10^−4^ mol·cm^−3^ and a lower crosslinking density for X-PBS_3, 2.01 × 10^−4^ mol·cm^−3^ having longer polymer chains between two consecutive crosslinking points. Indeed, the crosslinking density values determined by the theory of rubber elasticity are very similar to the ones calculated using the Flory–Rehner equation from the swelling experiments.

### 3.6. Mechanical Properties

The mechanical behavior of the systems was also investigated in tensile tests carried out above the melting temperature, to evaluate the material modulus and the ultimate properties.

The results are reported in Table 6 in terms of stress and strain at break and elastic modulus, evaluated from the slope of the stress-strain curve. While elongation at break does not show significant differences for the three materials, stress at break and the elastic modulus increase by increasing the network density and the silica content. The elastic modulus in the rubbery region evaluated by the tensile test is very similar to the storage modulus (E’_rubber_) evaluated with DMTA.

### 3.7. Shape Memory Behavior

The shape memory properties of the polymer networks were quantified by cyclic, stress-controlled thermo-mechanical tensile experiments. The strain fixity ratio, *R_f_*, quantifies the fixation of the temporary shape and is given by the ratio of the tensile strain after unloading, ε*_u_* (*N*), to the maximum strain at deforming stress (0.15 MPa) after cooling to *T*_low_, ε_l_ (*N*), for the *N*-th cycle, as detailed in the Experimental Section. The crystalline phase is used as a molecular switch unit to allow the transformation between the “temporary” and “permanent” shape, due to a change in chain mobility, allowing a macroscopical freezing of the temporary shape below *T_c_* (shape fixing) and an entropy-driven recovery processes above *T*_m_ (shape recovery). The strain recovery ratio, *R_r_*, quantifies how well the permanent shape has been memorized and is a measure of how far a strain applied in the course of the programming is recovered as a result of the shape memory effect [25]. The Si–O–Si crosslinks between the polymeric chains stabilize the permanent shape, allowing for the recovery process above the melting temperature. *R_f_* and *R_r_* of all the prepared networks are reported in Table 7. Comparing the shape memory properties of the first cycle with the ones of the second and third cycles, a remarkable improvement is observed in the recovery ratio values (*R_r_*). These changes in the first cycle are related to the thermo-mechanical history of the sample and can be mainly attributed to the viscous flow and/or plastic deformation of segments in the direction of deformation. For that purpose, the first cycle can be performed as a sort of preconditioning for the specimens before the subsequent cycles [31], with the aim of increasing the shape-memory performance, so in the rest of the paragraph, *R_r_* and *R_f_* values are discussed for *N* = 2 and *N* = 3. X-PBS_1 shows low strain fixity ratios, due to the very low crystallinity content, while X-PBS_2 and X-PBS_3 have very high values of *R_f_* (>99.1%), indicating an excellent capacity of the materials to fix the temporary shape. Concerning *R_r_*, the capacity to recover the permanent shape is related to the crosslinking density, since chain slippage and breakage decrease by increasing the network density (*R_r_* > 99% for X-PBS_1). Despite X-PBS_3 being the material with the lowest crosslinking density, it shows, however, a good value of *R_r_* (>87.8%). Significant differences between the second and third cycle for *R_f_* and *R_r_* are not present.

For X-PBS_2, the material showing the best shape memory properties, a second cyclic, stress-controlled thermo-mechanical tensile test was performed using a higher deforming stress (0.25 MPa). The results are summarized in Table 7. Even if a higher stress is applied, the material is still deformed in the elastic region, quite far from the yielding point, resulting in an excellent fixity capacity (*R_f_* > 98.7%) and even in a good recovery ability (*R_r_* > 96.7%).

The recovery rate, *ν_r_*, was calculated as the ratio of the strain recovery ratio, *R_r_*, over the temperature interval of recovery, Δ*T_r_*, determined as the difference between the temperature at which the recovery is completed (*T_e_*) and the temperature at which the recovery starts (*T_s_*) [32,33]:

$$\nu_{r}=\frac{R_{r}}{\Delta T_{r}}$$ (10)

$$\Delta T_{r}=T_{e}-T_{s}$$ (11)

The results, reported in Table 7, are averaged over the three cycles and show that the highest recovery rate was reached for X-PBS_2, due to both the high strain recovery ratio and the relatively narrow temperature interval of the strain recovery, while X-PBS_1, despite having a high value of *R_r_*, shows the lowest value of *ν_r_*, due to the wide Δ*T_r_*, related to a very broad melting transition. Indeed, for X-PBS_2, *ν_r_* does not show differences for the two applied stresses.

Besides the aforementioned strain fixity ratio and recovery behavior, also the energy stored after load removal in the programming cycle was taken into account to describe the performance of a shape memory system. The result was directly evaluated by integrating the nominal stress *vs.* nominal strain curves during the programming phase (*i.e.*, during deformation above *T_m_*, cooling under fixed stress and unloading below *T_c_*) as already described by Pandini *et al.* [13]. The results, reported in Table 7 are the average of the three cycles and show that under the application of the same load, a larger energy amount can be stored during the programming phase by the less crosslinked systems, due to their higher deformability and better capacity of fixing (higher degree of crystallinity). Considering the effect of the applied stress, higher stress corresponds to a larger stored energy, due to the higher level of strain attained during the deformation step.

Finally, it is noteworthy that the whole shape-memory cycle occurs at temperatures equal or higher than the room temperature, making it easy to fix a temporary shape by simply cooling at room temperature, which is below the crystallization temperatures for all the materials, and to activate the shape recovery by simply using hot air.

## 4. Conclusions

Crosslinked organic/inorganic hybrid semi-crystalline SMPs based on PBS were prepared using a water-induced silane crosslinking approach. This method permits one to obtain, under organic solvent-free and catalyst-free conditions, covalently crosslinked PBS-based SMPs, with Si–O–Si domains behaving as net-points necessary for shape memory behavior. Varying the PBS precursor molecular weight allows one to obtain materials with different crosslinking densities and different inorganic content, and, consequently, different properties and shape memory behaviors. The crosslinking density is seen to be an important parameter to control the shape memory behavior, since it governs both the melting temperature, which can be tuned in the range 89–101 °C, above which the recovery process is completed, and the crystallinity content, which defines the fixity capability. The results show that through an appropriate chemical tuning of the molecular structure of the polymer network, it is possible to obtain materials with a very good shape memory behavior. Promising features for shape memory applications are implied, because the whole cycle occurs at temperatures above room temperature, which makes it easy to fix the temporary shape and to activate the shape memory behavior by simply using hot air.

## Figures and Tables

**Table 1.** Molecular weights (*M_n_*) of α,ω-hydroxyl-terminated PBS and the degree of silanization (*D_SI_*) of α,ω-triethoxysilane-terminated PBS as determined by ^1^H NMR spectroscopy.

Code	*M_n_* (g·mol^−1^)	Code	*D_SI_* (%)
PBS_1	1400	PBS_1_Si	98
PBS_2	2200	PBS_2_Si	91
PBS_3	4400	PBS_3_Si	92

**Table 2.** Gel content (*G*), degree of swelling (*Q*), crosslinking density and average molecular weight between crosslinks (*M_c_*) of the crosslinked PBSs.

Code	*G* (%)	*Q*	Crosslinking density (mol·cm^−3^)	*M_c_* (g·mol^−1^)
X-PBS_1	99.2	4.9	9.44 × 10^−4^	1350
X-PBS_2	91.5	7.1	4.60 × 10^−4^	2750
X-PBS_3	95.5	9.8	2.52 × 10^−4^	5000

**Table 3.** Thermogravimetric analysis: actual silica content (mass residue at 750 °C) and the maximum decomposition temperature (*T*_max_) of X-PBS_x.

Code	Residual weight (%)	*T*_max_ (°C)
X-PBS_1	14.1	410
X-PBS_2	10.1	411
X-PBS_3	4.9	399

**Table 4.** Thermal properties (crystallization temperature, *T_c_*; melting temperature, *T_m_*; degree of crystallinity, α of linear PBS (PBS_x), alkoxysilane-terminated PBS (PBS_x_Si) and crosslinked PBS (X-PBS_x) determined by DSC.

Code	*T_c_* (°C)	*T_m_* (°C)	α (%)
PBS_1	69	107	46.9
PBS_1_Si	64	102	32.0
X-PBS_1	–	–	–
PBS_2	74	113	45.8
PBS_2_Si	70	111	39.8
X-PBS_2	51	89	17.2
PBS_3	77	114	46.8
PBS_3_Si	73	113	41.2
X-PBS_3	64	101	21.3

**Table 5.** Glass transition (*T_g_*), damping (tanδ_max_) and storage moduli at different temperatures (−50 °C, 27 °C and in the rubbery plateau) and crosslinking densities (*νe*) of crosslinked PBSs.

Code	*T*_g_ (°C)	tanδ_max_ (−)	E’_−50°C_ (GPa)	E’_27°C_ (MPa)	E’_rubber_ (MPa)	*ν_e_* (mol·cm^−3^)
X-PBS_1	−8	0.217	1.6	87	8.2	8.76 × 10^−4^
X-PBS_2	−13	0.152	2.6	337	4.4	4.23 × 10^−4^
X-PBS_3	−14	0.143	1.9	300	2.2	2.01 × 10^−4^

**Table 6.** Results of tensile tests carried out in the rubbery region.

Code	Stress at break (MPa)	Elongation at break (%)	Elastic modulus (MPa)
X-PBS_1	1.1 ± 0.1	20 ± 1	7.7 ± 1.6
X-PBS_2	0.5 ± 0.2	28 ± 9	3.3 ± 1.6
X-PBS_3	0.2 ± 0.1	18 ± 12	3.0 ± 0.4

**Table 7.** The strain fixity (*R_f_*) and strain recovery (*R_r_*) ratios for the *N*-th cycle (*N* =1, 2 and 3), the interval temperature recovery (Δ*T_r_*), the recovery rate (*ν_r_*) and the stored energy for crosslinked PBSs at different applied stresses.

Applied stress (MPa)	Code	*N* = 1		*N* = 2		*N* = 3		Δ*T_r_* (K)	*ν_r_* (% K^−1^)	Stored energy (kJ·m^−3^)
		*R_f_* (%)	*R_r_* (%)	*R_f_* (%)	*R_r_* (%)	*R_f_* (%)	*R_r_* (%)			
0.15	X-PBS_1	78.9	58.9	83.9	100.0	80.3	99.0	24	3.6	0.17
0.15	X-PBS_2	99.2	77.9	99.3	91.1	99.2	91.9	10	8.7	2.23
0.15	X-PBS_3	98.7	74.9	99.2	87.8	99.1	89.4	16	5.3	3.81
0.25	X-PBS_2	98.6	70.4	98.7	96.7	98.7	96.9	10	8.8	11.7

## Supplementary Materials

Supplementary File 1
