# Supplementary material for: Shape Memory Properties of PBS-Silica Hybrids
Source: Materials (Basel). 2014 Jan 27;7(2):751–68. doi: 10.3390/ma7020751 (PMC5453071; doi:10.3390/ma7020751)
Supplement: Supplementary File 1 [file materials-07-00751-s001.pdf]

Supporting Information

Determination of the Crosslinking Density of PBSs Using the Flory–Rehner Equation

Crosslinking density, defined as moles of effective network chain per cubic centimeter, was computed from equilibrium swelling of the networks in $CHCl_3$, calculating the volume fraction of the swollen polymer [1,2]. For this, the swell ratio (Q') was obtained experimentally by placing specimens having a rectangular shape (a length of 20 mm and a cross-section of 5×1 mm^2) and initial weight m_o in $CHCl_3$, a solvent in which PBS is soluble.

In this solvent, the polymeric chains that are not crosslinked are solubilized by $CHCl_3$, while the three-dimensional network polymer swells, absorbing a large quantity of the solvent with which it is placed in contact [3]. As the network is swollen absorbing $CHCl_3$, the chains between crosslinking points are required to assume elongated configurations, and a retroactive force develops in opposition to the swelling process, until a state of equilibrium swelling is reached.

After approximately 24 h, estimated as the time sufficient for reaching equilibrium swelling, the specimens were removed from the solvent, and the weight of the swollen specimen was determined (m_s). The swollen specimens were subsequently dried in order to determine the residual weight after extraction (m_d):

$$Q' = \frac{m_s}{m_d} - 1 \quad \text{(S1)}$$

The weight fraction of the polymer (W_2), normalized on the organic phase content, and the solvent (W_1) can then be calculated by the relation:

$$W_2 = \frac{1}{1+Q'} \quad \text{(S2)}$$

and:

$$W_1 = 1 - W_2 \quad \text{(S3)}$$

The volume fraction of the polymer in the swollen matrix (υ_2) is given by:

$$\upsilon_2 = \frac{W_2/\rho_2}{W_2/\rho_2 + W_1/\rho_1} \quad \text{(S4)}$$

where ρ_1 and ρ_2 are the densities of the solvent and the polymer, respectively.

The crosslink densities (ν) of the polymer networks, in which the junctions are f-functional, were obtained from υ_2 using the Flory–Rehner relation [3]:

$$\nu = \frac{-\left[\ln(1-\upsilon_2) + \upsilon_2 + \chi_{1-2}\upsilon_2^{\,2}\right]}{V_s\left(\upsilon_2^{1/3} - \frac{2\upsilon_2}{f}\right)} \quad \text{(S5)}$$

where V_s is the molar volume of the solvent (80.70 cm^3 mol^{-1} for chloroform) and χ_{1-2} is the Flory–Huggins interaction parameter between the polymer and the solvent [2,4]. The polymer-solvent interaction parameter, χ_{1-2}, can be calculated according to:

$$\chi_{1-2} = \frac{(\delta_1 - \delta_2)^2 V_s}{RT} \tag{S6}$$

where δ_1 and δ_2 are the solubility parameters of the solvent and polymer, respectively. *R* is the gas constant (8.314 J K^{-1} mol^{-1}), T the absolute temperature (298 K), δ_1 of chloroform is 18.95 MPa$^{1/2}$ [5], while δ_2, which can be calculated by several methods involving group contributions, was determined by the Van Krevelen approach [6], one of the most common methods in which each parameter can be estimated using the following equations (Van Krevelen and Hoftyzer, 1976):

$$\delta_d = \frac{\sum F_{di}}{V} \tag{S7}$$

$$\delta_p = \frac{\sqrt{\sum F_{pi}^2}}{V} \tag{S8}$$

$$\delta_h = \sqrt{\frac{\sum E_{hi}}{V}} \tag{S9}$$

where *V* is the molar volume of the polymer determined with the additive group method, δ_d, δ_p and δ_h are the contributions of the dispersion forces, polar forces and hydrogen bonding forces to the solubility parameter, respectively, F_{di} is the dispersion component and F_{pi} the polar component of the molar attraction function and E_{hi} is the contribution of the hydrogen bonding forces to the cohesive energy for the *i*-th group.

The total solubility parameter is then calculated by:

$$\delta_2 = \sqrt{\delta_d^2 + \delta_p^2 + \delta_h^2} \tag{S10}$$

References

1. Jain, S.; Sekkar, V.; Krishnamurthy, V. Mechanical and swelling properties of HTP-based copolyurethane networks. *J. Appl. Polym. Sci.* **1993**, *48*, 1515–1523.
2. Bordes, C.; Fréville, V.; Ruffin, E.; Marote, P.; Gauvrit, J.; Briançon, S.; Lantéri, P. Determination of poly(ε-caprolactone) solubility parameters: Application to solvent substitution in a microencapsulation process. *Int. J. Pharm.* **2010**, *383*, 236–243.
3. Flory, J. P. *Principles of Polymer Chemistry*; Cornell University Press: Ithaca, NY, USA, 1953.
4. Ma, P.; Ma, Z.; Dong, W.; Zhang, Y.; Lemstra, P.J. Structure/property relationships of partially crosslinked poly(butylene succinate). *Macromol. Mater. Eng.* **2013**, *298*, 910–918.
5. Hansen, C.M. *Hansen Solubility Parameters: A User's Handbook*, 2nd ed.; CRC Press: Boca Raton, FL, USA, 2007.
6. Van Krevelen, D.W. *Properties of Polymers*; Elsevier: New York, NY, USA, 1990.
